# Supplementary material for: Publication bias and the tourism-led growth hypothesis
Source: PLoS One. 2021 Oct 14;16(10):e0258730. doi: 10.1371/journal.pone.0258730 (PMC8516250; doi:10.1371/journal.pone.0258730)
Supplement: Attachment — Submitted filename: 2_PLOS1_Response to Reviewers.docx [file pone.0258730.s003.docx]

**Response to Editors and Reviewers Comments**

We would like to thank the handling academic Editor of PLOS ONE, Professor Hiranya Nath, and the anonymous reviewers for accepting the changes we had made in the earlier version of the manuscript. We would also like to thank the journal’s editorial team for efficiently moving the paper through the peer review process. We sincerely appreciate that the reviewers are satisfied with the changes made. The improvements would not have been possible without their useful comments.

We have checked and updated the references list and ensured that (1) the reference list is complete, and (2) we have not cited any retracted articles in our study consistent with the journals requirements.

We have summarized the changes in the Table below:

| **Comment** | **Description of Changes** |
| --- | --- |
| 1) p. 6, line 123: delete one of the two “been”s | This has been amended. See line 123 in the updated manuscript |
| 2) p. 6, line 140: expand PIC as you are using it for the first time | This has been amended. The first instance of Pacific Island Countries was in line 125. PIC is defined in line 125 instead of line 140. |
| 3) p. 7, line 151: use ‘adapted’ instead of ‘adopted’ | This has been amended. Please see line 151 in the updated manuscript. |
| 4) Include data sources in References and cite appropriately | These are updated and amended. Citations are included from line 185 to 189 in the updated manuscript. The reference section is amended accordingly. These citations are entries 30 to 33. The original citations are amended accordingly. Guidance is sought from the journals reference style guidelines: <https://journals.plos.org/plosone/s/submission-guidelines#loc-references>  Please see the updated manuscript with track changes to review these changes. |
| 5) p. 9: Give a very brief explanation on why you convert data frequency | This has been updated. Please see lines 197-198 and 205-206 for these changes. |
| 6) Include the augmented terms (differenced y lags) in Eq. (4) | Eq. (4) is updated to reflect these changes. Please see line 211 for the updated Eq. (4). |
